# Online, real-time detection of volatile emissions from plant tissue

**DOI:** 10.1093/aobpla/plt003

**Published:** 2013-01-09

**Authors:** Frans J. M. Harren, Simona M. Cristescu

**Affiliations:** Life Science Trace Gas Facility, IMM, Radboud University, Heyendaalseweg 135, 6525 AJ Nijmegen, The Netherlands

**Keywords:** Ethylene, laser-based detection, nitric oxide, plant volatiles, proton transfer reaction mass spectrometry, real-time emission, trace gas detection, volatile organic compounds

## Abstract

Trace gas monitoring plays an important role in many areas of life sciences ranging from agrotechnology, microbiology, molecular biology, physiology, and phytopathology. In plants, many processes can be followed by their low-concentration gas emission, for compounds such as ethylene, nitric oxide, ethanol or other volatile organic compounds (VOCs). For this, numerous gas-sensing devices are currently available based on various methods. Among them are the online trace gas detection methods; these have attracted much interest in recent years. Laser-based infrared spectroscopy and proton transfer reaction mass spectrometry are the two most widely used methods, thanks to their high sensitivity at the single part per billion level and their response time of seconds. This paper starts with a short description of each method and presents performances within a wide variety of biological applications. Using these methods, the dynamics of trace gases for ethylene, nitric oxide and other VOCs released by plants under different conditions are recorded and analysed under natural conditions. In this way many hypotheses can be tested, revealing the role of the key elements in signalling and action mechanisms in plants.

## Abstract (precis)

Using sensitive and real-time detection of volatiles from plants with state-of-the-art laser based- and mass spectrometry-based methods many, hypotheses can be tested, revealing the role of the key elements in signalling and action mechanisms in plants.

## Introduction

Reliable monitoring of small quantities of trace gases in complicated gas mixtures is of great importance for all research areas of life sciences. Atmospheric chemists are interested in the origin of air pollution and its consequences, such as the global greenhouse warming or the depletion of the ozone layer. Medical researchers are searching for volatile exhaled biomarkers for non-invasive detection and treatment monitoring of various diseases. Biologists would like to know the physiological status of plant material, and how it is reacting in biotic and abiotic stress situations, such as drought, flooding, high/low temperatures, herbivore attack, fungal infestation, etc. Quality assurance in food production (e.g. transport and storage) may improve its quality at reduced costs and ensure the safety of the consumers. In most of these situations, *in vivo*, online and non-invasive monitoring of trace gases is desired. Nowadays, laser-based and mass spectrometry-based methods can routinely detect trace gas quantities down to 1 part per billion by volume (ppbv; 1:10^9^) or lower at a timescale of seconds (Fall *et al*. 1999; Kosterev and Tittel 2002; Brown 2003; Tittel *et al*. 2003; Tholl *et al*. 2006; Cristescu *et al*. 2008; Loreto and Schnitzler 2010).

To study trace emissions of molecules produced by biological samples, highly sensitive detectors are required, owing to the sometimes very low emission rate or small amount of plant material available. A fast response time is often required for accurate monitoring of the dynamics of the physiological process. The type and number of molecules emitted vary as much as their sources. Molecules from a wide range of volatile organic compounds (VOCs) can be released: alkanes, alkenes, alcohols, aldehydes, ketones, acids, esters, aromatics, halogenated hydrocarbons, sulfur- and nitrogen-containing compounds. Vapours can also be measured from the liquid phase; the necessary condition for the emission in gas phase is that at a specific temperature the vapour pressure should be sufficiently high. This is almost always the case, since the ppmv/ppbv vapour pressure level of the majority of gases is far below 0 °C (Lide 1993).

Many different methods have been developed for trace gas detection, ranging from electronic noses, infrared spectrometers, laser-based detectors to mass spectrometers, whether or not combined with gas chromatography. Some of the trace gas detectors are specialized on a specific molecule, resulting in highly sensitive detectors; others have a much more analytical approach, measuring many different compounds simultaneously. Here, we will consider the two most widely used approaches for sensitive online trace gas detection: laser-based infrared spectroscopy and mass spectrometry. A short description of laser-based spectroscopic methods and sensitive mass spectrometry is given, with emphasis on why these approaches are so selective and sensitive for the detection of trace gases. This brief technological approach is followed by a number of examples focused on specific applications and molecules related to plant physiological processes, such as ethylene, ethane, nitric oxide (NO), ethanol, acetaldehyde, etc.

## Laser-based trace gas detection

**Fig. 1:**
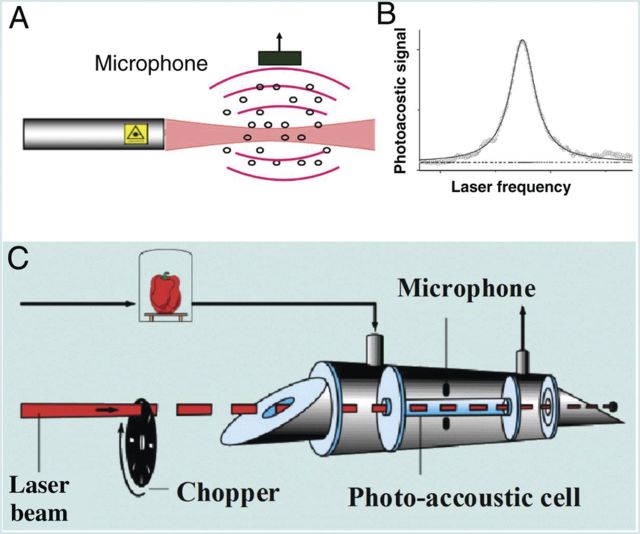
**Laser-based photoacoustic trace gas detection.** (A) Generation of photoacoustic sound, (B) laser frequency scan over the absorption line of a molecule and (C) schematic view of an experimental set-up. When a gas molecule absorbs laser radiation, the molecule is excited to a higher quantum state. De-excitation of that level under atmospheric pressure by collisions causes the release of energy in the form of heat. In a closed volume this causes a pressure increase. When the light beam is modulated with a chopper at an audio frequency (C, e.g. 1 kHz), a pressure wave will be generated: sound will appear at a frequency of 1 kHz. The strength of the sound will be equal to the absorption strength (gas concentration), while the laser frequency gives the selectivity to distinguish the gas in a mixture of gases.

A gaseous molecule that absorbs light/laser radiation is excited to a higher quantum state. This absorption causes a decrease in laser light intensity, which can be directly quantified via absorption spectroscopy (Werle 1998). More indirect methods are observing the depopulation of the excited state via fluorescence (i.e. fluorescence spectroscopy) or by collisional de-excitation. The latter gives rise to a temperature and pressure change in the gas that can be detected with a sensitive microphone (photoacoustic spectroscopy) (Fig. 1) (Miklos *et al*. 2001; Harren *et al*. 2012).

In general, laser spectroscopy is very specific; a molecular transition (and thus absorption frequencies of the light) can be determined with very high accuracy. Each molecular gas has thousands of specific absorption lines. For this reason, the infrared wavelength region between 2 and 20 μm is called the fingerprint region; the molecule gives a unique, specific absorption pattern that can be clearly discriminated from other gases. On the other hand, if there is a complex mixture of gases, care has to be taken to choose a proper absorption line for the determination of the concentration, where there should be low or no interference with absorption lines of other gases (Rothman *et al*. 2009).

Next to its specificity, laser spectroscopy can achieve a very high sensitivity. Within absorption spectroscopy this is done by increasing the path length of the light through the gas. For this, mostly an absorption cell is used with high reflective mirrors in a multipass arrangement, combined with advanced modulation techniques. This type of absorption spectroscopy has evolved in a wide range of methods such as cavity enhanced spectroscopy, cavity ring down spectroscopy, wavelength modulation spectroscopy, etc.; an overview of these spectroscopic methods can be found elsewhere (Berden *et al*. 2000; Brown 2003). Alternatively, photoacoustic spectroscopy does not need a long absorption path length, due to its intrinsic high sensitivity with high laser power (Bijnen *et al*. 1996; Harren *et al*. 2012). Moreover, it is a simple technique that can be used in compact and robust schemes for online trace gas detection (Cristescu *et al*. 2008).

**Fig. 2:**
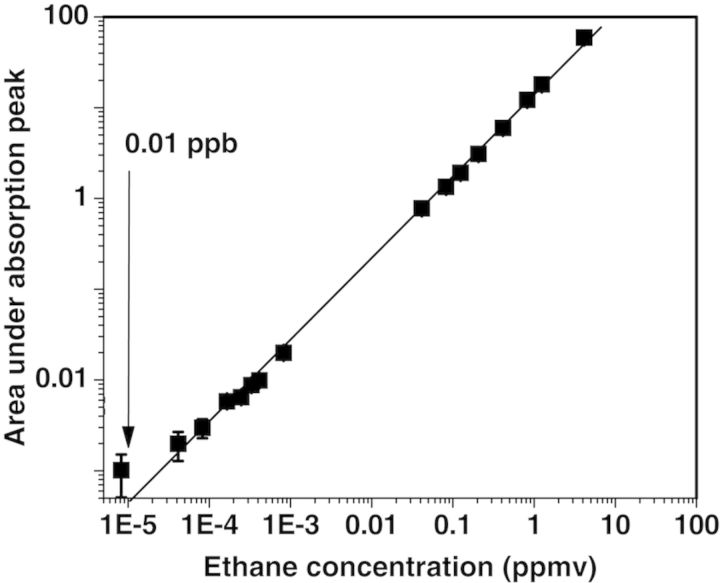
**Linearity of the laser-based photoacoustic system.** The measured signal is displayed vs. the applied gas concentration over >6 orders of magnitude for ethane (van Herpen *et al*. 2002).

To quantify gas concentrations, it is important to calibrate the sensor with a known gas mixture and to show the linearity of the detector signal with the concentration of the probed gas over orders of magnitude. As an example, the linear response of the photoacoustic sensor for low detection limits of ethane is presented in Fig. 2 (van Herpen *et al*. 2002).

## Proton transfer reaction mass spectrometry

Using mass spectrometry, electron ionization typically results in extensive fragmentation of the molecule under investigation, which can be an advantage for identifying single compounds. However, the interpretation of the mass spectra (i.e. the confirmation of molecular weight) can be difficult when only traces of a gas are available in complex gas mixtures, such as air. Fragmentation should then be avoided. One way is via soft chemical ionization; examples of this are ion mobility mass spectrometry (Kanu *et al*. 2008), selective ion flow tube mass spectrometry (Smith and Spanel 2005) or proton transfer reaction mass spectrometry (PTR-MS) (Lindinger *et al*. 1998; de Gouw *et al*. 2003).

Within PTR-MS, molecules typically form a protonated molecular ion [M + H]^+^, with M being the molecular weight of the parent molecule. Next to its low fragmentation rate, PTR-MS has the advantage of being very efficient, resulting in very good performance for online trace gas detection. The strength of this technique is detection of trace gases from various chemical groups in the order of seconds at (sub)part per billion levels. In comparison, when using gas chromatographic methods the analytic capabilities are strongly enhanced, but the time response is increased to 20–30 min. Therefore, PTR-MS is best used with online, real-time experiments when a fast time response is expected, or when a number of samples are monitored in parallel.

**Fig. 3:**
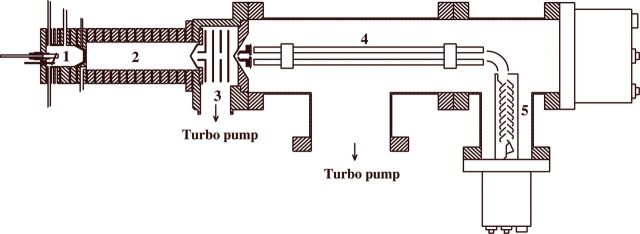
**Schematic view of the PTR-MS instrument.** (1) The discharge source for the production of H_3_O^+^ ions; (2) the drift tube: the trace gases from the biological sample enter here and a proton transfer takes place with the ions from chamber (1); (3) the buffer chamber; (4) the mass spectrometer with quadrupole; and (5) the secondary electron multiplier.

The working principles of PTR-MS have been given in detail elsewhere (Lindinger *et al*. 1998; de Gouw *et al*. 2003; Boamfa *et al*. 2004; Blake *et al*. 2009) (see Fig. 3). The instrument consists of four parts: an ion source where H_3_O^+^ ions are produced, a drift tube section, a separately pumped transition chamber, and an ion detection section containing a quadrupole mass spectrometer and a secondary electron multiplier. In the drift tube, the trace gases from the sample gas are ionized by proton-transfer reactions with H_3_O^+^ ions: H_3_O^+^ + R → RH^+^ + H_2_O. This reaction only takes place if the proton affinity (PA) of the trace compound R is higher than that of water (166.5 kcal/mol = 7.16 eV per molecule). A major advantage of using H_3_O^+^ as the reagent ion is that the PA of water is higher than the PA of the normal constituents of air: N_2_, O_2_, CO_2_, CH_4_, CO, NO and argon, and most of the typical organic compounds are ionized by the proton-transfer reaction, since their PA are in the range between 7 and 9 eV. The reaction rate can be measured or calculated and is known for many of the proton-transfer reactions of interest (Hunter and Lias 1998). Since the excess energy of the reaction is low, it results in only one or two characteristic ions per neutral molecule. For some molecular groups, dissociation can occur to form one or two fragments of significant intensity (e.g. alcohols can split off a water molecule). Owing to this soft ionization the matrix of signals is less complicated than with other mass spectrometric techniques. However, a drawback is that the compounds cannot always be identified uniquely, as each detected mass can sometimes be associated with more than one compound or compound fragment.

## Detection of the plant hormone ethylene

One of the first and most successful applications of laser-based trace gas detection in biology is detection of ethylene (C_2_H_4_). Ethylene is a plant hormone that plays an important role in the regulation of many environmentally and developmentally induced processes, such as stress resistance, germination, ripening, senescence and abscission (Yang and Hoffman 1984). Ethylene production can also be induced by a variety of external factors such as mechanical wounding, environmental stresses and interaction with various micro-organisms. All tissue types and probably all cells of higher plants produce and liberate ethylene (Bleecker and Kende 2000). Many lower plants such as liverworts, mosses, ferns, lycopods and horse tails also are producers of ethylene, although the biosynthetic route seems to be different (Osborne *et al*. 1996). Ethylene is biosynthesized from the amino acid methionine to *S*-adenosyl-l-methionine (SAM) by the enzyme methionine adenosyltransferase. *S*-adenosyl-l-methionine is then converted into 1-aminocyclopropane-1-carboxylic acid (ACC) by the enzyme ACC synthase (ACS) (Yang and Hoffman 1984). The activity of ACS determines the rate of ethylene production, and therefore regulation of this enzyme is key for ethylene biosynthesis. The final step to ethylene requires oxygen and involves the action of the enzyme ACC oxidase. Environmental stresses (physical, chemical and biological) and hormonal signals, such as auxin, cytokininin and even ethylene itself, stimulate the synthesis of the ACC synthase, thereby providing a means for autoregulation of its production (Ecker 1995).

With laser-based photoacoustic spectroscopy, ethylene can be detected with high sensitivity, high speed and very good selectivity. The use of this laser-based detector in combination with a flow-through system is proven to be unbeatable in sensitivity and time response in comparison with traditional methods such as gas chromatography, which is currently most widely used (Woltering *et al*. 1988; Bijnen *et al*. 1996; Cristescu *et al*. 2008). Briefly, traces of ethylene released by various biological samples absorb laser radiation inside the photoacoustic cell. The ethylene concentration is calculated from a comparison of the photoacoustic signals on various laser emission frequencies (at which ethylene has different absorption strengths). The laser-based instrument allows detection of ethylene emission in a continuous-flow system down to 0.01 ppbv over 90 s (Bijnen *et al*. 1996). In recent years, the laser-based ethylene detection system has been used within the Life Science Trace Gas Facility (www.ru.nl.tracegasfacility) for online measurement of ethylene in various dynamic processes in plants and micro-organisms.

One of the first and most appealing applications is detection of ethylene emission from flowers during their development, pollination and senescence. Owing to their low mass (∼1 g) and sensitivity to ethylene they emit only small amounts of ethylene (nL h^−1^). The ethylene emission could be observed at a very early stage, as was shown with orchid (*Cymbidium* cv. Mary Pinchess ‘Del Rey’) flowers after emasculation (removal of the pollinia plus anther cap) (Woltering *et al*. 1988). Within 3 h, an increase in ethylene emission could be observed, well before colouration of the labellum (after 8 h) or wilting of the petals and sepals (45 h). In a later study, it was shown that desiccation of the rostellum is responsible for post-emasculation phenomena in orchid flowers (Woltering and Harren 1989*b*). Studies on early changes in ethylene production during senescence of other orchid, carnation and petunia flowers followed (Woltering and Harren 1989*a*; Woltering *et al.* 1993) and in a later study pollination and stigma wounding were compared in petunia flowers (Woltering *et al*. 1997). In flowers of *Nicotiana tabacum* L., pollination induces a transient increase in ethylene production by the pistil (De Martinis *et al*. 2002). The characteristic ethylene emission dynamics correspond to the pollen-tube journey into the pistil: penetration into the stigma, growth through the style, entry into the ovary and fertilization; it was shown that ethylene is synthesized *de novo* in the pistil.

Senescence of floral organs can be broadly divided into two groups: those that exhibit sensitivity to exogenous ethylene and those that do not (Wagstaff *et al*. 2005). Endogenous ethylene production by the former group is via a well-characterized biochemical pathway and is due either to developmental or pollination-induced senescence. Flowers from the ethylene-insensitive group do not appear to produce endogenous ethylene, or respond to exogenous ethylene treatments. From this group the role of ethylene in the senescence and abscission of *Alstroemeria peruviana* cv. Rebecca and cv. Samora tepals was investigated (Wagstaff *et al*. 2005). Results indicate that *Alstroemeria* is clearly sensitive to extremely small concentrations of endogenous and exogenous ethylene, although sensitivity develops late in the life of this flower.

**Fig. 4:**
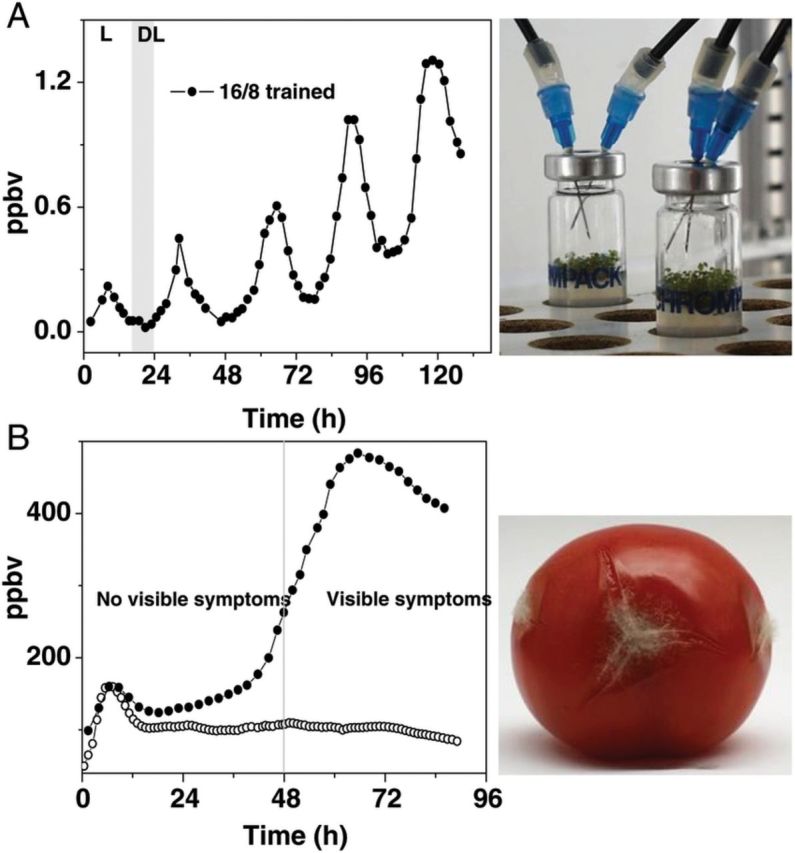
**Ethylene production from various biological samples.** (A) Plant physiology: circadian clock in *A. thaliana* with a peak in the mid-subjective day (Thain *et al*. 2004); (B) early detection of *B. cinerea* (grey mould) infection in tomato fruits. Real-time production by artificially inoculated tomato fruits (at *t* = 0 h) with *B. cinerea* (filled circles) starts rising 1 day before visible infection as compared with uninfected fruit injected with water (open circles) (Cristescu *et al*. 2002).

Other processes that have been monitored online are seed germination (Gianinetti *et al*. 2007), fruit ripening (Iannetta *et al*. 2006), circadian rhythm in plants (Fig. 4A) (Thain *et al*. 2004), plant–pathogen interaction (Fig. 4B) (Cristescu *et al*. 2002; Montero *et al*. 2003), plant response to insect egg deposition (Schroder *et al*. 2007), interaction with other plant hormones (Vandenbussche *et al*. 2003*a*, *b*; Clarke *et al*. 2009; Roeder *et al*. 2009), dehydration and drought (Leprince *et al*. 2000; Balota *et al*. 2004), flooding (Voesenek *et al*. 1990, 1993) and programmed cell death (Yakimova *et al*. 2006). Based on this technology, a simple, robust and easy to maintain ethylene detector has been developed (Sensor Sense BV, Nijmegen, The Netherlands), and a number of the above-described studies have been performed with this commercial device. It achieves a noise equivalent minimum detection limit of 0.07 ppbv within a 5-s observation time (De Gouw *et al*. 2009).

## Detection of methane isotope emission from plants

The detection of trace gases using laser-based detectors depends strongly on the overlap of the available wavelength region from the laser and the wavelength of the molecular absorption line. For the detection of methane, the 3-μm infrared wavelength region is the most favourable (Rothman *et al*. 2009). Recently, optical parametric oscillators (OPOs) were developed for this wavelength region to detect trace gases using photoacoustic spectroscopy (Ngai *et al*. 2006, 2007). An excellent detection limit of 0*.*01 ppbv for ethane could be obtained in 40 s (van Herpen *et al*. 2002). In addition, this OPO system was used to monitor ^12^CH_4_ and ^13^CH_4_ emissions by plants, for which the system has a detection limit of 0*.*1 ppbv for both isotopes. The study of methane emissions from plants was provoked by an earlier study published by Keppler *et al*. (2006) which found that terrestrial plants can produce a large amount of methane in aerobic conditions. In collaboration with other groups, we re-examined this finding using an independent test (Dueck *et al*. 2007). For this study, ^13^C-labelled plants grown under controlled conditions in an Experimental Soil Plant Atmosphere System (ESPAS; IsoLife BV, Wageningen, The Netherlands) (Gorissen *et al*. 1996) were used. When investigating possible methane emission, it is preferable to detect ^13^CH_4_ from plants, since the natural atmospheric background concentration of ^13^CH_4_ is only 20 ppbv, compared with a high level of 1*.*7 ppmv for ^12^CH_4._ The ESPAS is a unique, hermetically sealed, plant growth chamber (3*.*5 m^3^), specifically designed for growing full ^13^C-plants, applying ^13^CO_2_ in its atmosphere. For this research, various plant species (*Ocimum basilicum*, basil; *Salvia officinalis*, sage; *Triticum aestivum*, wheat; *Zea mays*, maize) were grown from seeds in hydroponics for a 9-week period in a ^13^CO_2_ atmosphere (99 % ^13^C, 1 % ^12^C), instead of a natural ^12^CO_2_ atmosphere (1*.*1 % ^13^C, 98*.*9 % ^12^C). Since ∼99 % of the carbon found in these plants was in the form of ^13^C, we can expect that nearly 99 % of the methane emitted by these plants is in the form of ^13^CH_4_. At the natural background concentration (20 ppbv), an accuracy of 3 ppbv for ^13^CH_4_ could be obtained in 60 s (Ngai *et al*. 2006).

For the methane emission experiments, the plants were transferred from the ESPAS to a continuous-flow gas exchange cuvette and analysed for their ^13^CH_4_ emission. We found that ^13^C-methane concentrations in the cuvettes with plants were not significantly higher than those in control cuvettes without plants; the difference was close to the detection limit of the system. Based on this difference, the emission rates for the four species ranged from 10 to 42 ng g^–1^ h^–1^, with an overall mean of 21 ng g^–1^ h^–1^; this is 6–18 times lower than the emission rates published earlier (Keppler *et al*. 2006). To improve the accuracy, a second experiment was performed; a large number of plants from six species (*O. basilicum* L., basil; *T. aestivum* L., wheat; *Z. mays* L., maize; *S. officinalis* L., sage; *Lycopersicon esculentum* Miller, tomato; *Oenothera biennis* L., common evening primrose) were grown in the ESPAS facility and the air was accumulated over a 6-day period. An increase over time of <1 ppbv ^13^CH_4_ was found, which implies an emission rate of between 0.9 and 0.4 ng g^–1^ h^–1^, and 0.1–0.3 % of the expected emissions based on earlier data (Keppler *et al*. 2006). Additional tests were performed to eliminate the possibility of eventual ^13^CH_4_ loss because of leakage or oxidation which showed neither of these. The results from these tests indicated that plants produce an insignificant amount of methane in aerobic conditions.

**Fig. 5:**
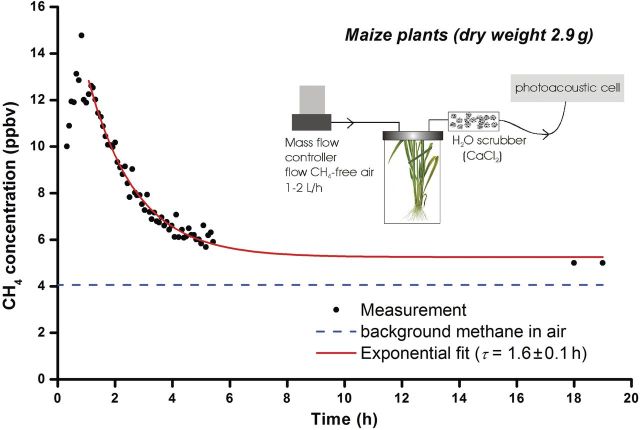
**Diffusion of atmospheric ^12^C-methane trapped in the plant cells.** Before the start of the experiment the plant cell was flushed with methane-free air for 1 h at 60 L h^−1^. After the initial measurements the flow was 2 L h^−1^. The water content was reduced by inserting a CaCl_2_ scrubber in the flow towards the photoacoustic cell.

We have also made online measurements of ^12^CH_4_ from ‘normal’ plants (not ^13^CH_4_) placed in closed cuvettes and flushed with methane-free air before starting the experiment as well as during it. The only methane recorded (Fig. 5) was due to the flushing out of the atmospheric methane trapped in the plant cells for several hours. This could explain, in part, the overestimated methane reported in Keppler *et al*. (2006) as production instead of atmospheric methane diffusion from plant tissue.

## Plant–pathogen interaction

After the first report of exhaled NO in animals and humans (Stewart *et al*. 1995), the interest in NO monitoring has increased. In plants, NO is a signalling molecule that plays an important role in plant growth and development, stomatal regulation, and protection against biotic and abiotic stresses (Besson-Bard *et al*. 2008; Wilson *et al*. 2008; Mur *et al*. 2011). The last decade has brought many new insights into pathways of NO production and function in plants, but many details have still to be elucidated. Nitric oxide, together with reactive oxygen intermediates, play a key role in the activation of disease resistance mechanisms (Delledonne *et al*. 1998). Plant diseases are a major source of crop loss worldwide and it is important to understand how plant defence mechanisms can neutralize infections. The most visible type of plant defence is the elicitation of a highly localized programmed cell death known as the hypersensitive response (HR). Nitric oxide has emerged as an important component of HR-associated defences, so that if its production is reduced, plant resistance may be compromised.

In 2005, a laser-based photoacoustic system was used for the first online, *in planta*, monitoring of the NO production from pathogen-infected tobacco leaves (Mur *et al*. 2005*b*). The conclusion of this work indicated that NO influenced the kinetics of cell death and resistance to both avirulent and virulent bacteria in tobacco, and suggested that NO is integral to the elicitation of cell death associated with these two bacterial pathogens in tobacco.

Detection of gaseous NO with chemiluminescence devices is considered the ‘gold standard’, providing sufficient accuracy and precision. A convenient alternative is offered by electrochemical sensors. In spite of their lower cost and the possibility for development of portable devices, electrochemical sensors suffer from a lack of sensitivity (detection limit >5 ppbv) (Mur *et al*. 2011). Chemiluminescence detectors demand high gas flow rates (>10 L h^−1^), which make these systems unsuitable for the detection of a low amount of NO emission. Within laser-based technology, which has the same sub-ppbv detection level as chemiluminescence, low gas flow rates over the plant tissue are not a problem (Cristescu *et al*. 2008; Mur *et al*. 2011).

**Fig. 6:**
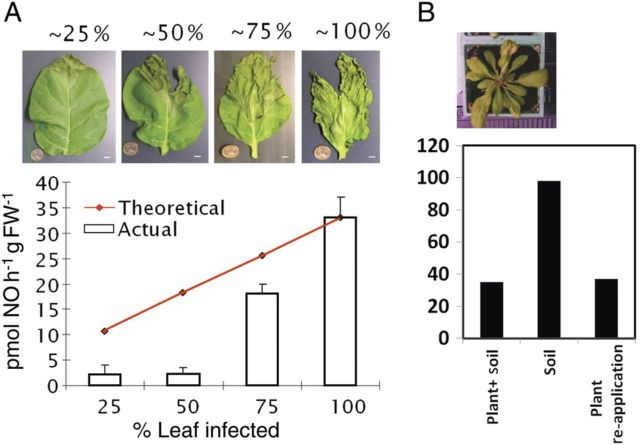
**Reduced NO detection with the inclusion of non-infected plant tissue (A) and soil (B).** (A) Nitric oxide production from tobacco leaves inoculated to a percentage level of the leaf with *Pseudomonas syringae* bacteria 6 h after the challenge. As can be seen, the actual NO production levels are considerably lower, as could be expected from the damage percentage of the leaf tissue, most probably due to oxidation of NO produced by infected tissue by the surrounding uninfected tissue. (B) Nitric oxide production from a 5-week-old *Arabidopsis* plant grown in soil, from the same soil after cutting the plant and reapplication of the cut rosette to the soil surface (Mur *et al*. 2011).

In standard plant experiments, isolated pathogen-challenged leaves are placed in a closed glass cuvette and flushed with air so that NO released in the headspace is transported to the laser-based system. However, the NO production following pathogen inoculation of intact plants (including the pot) is mainly due to the soil and roots (Fig. 6). When the plant was excised at the roots and removed, a higher NO emission from the soil was observed. Returning the excised plant to the cuvette reduced the NO emission again. This pattern shows that leaf tissues have the capacity to remove NO from the atmosphere (Fewson and Nicholas 1960; Mur *et al*. 2011). Therefore care must be exerted to make sure that as much as possible of the plant material under assessment is producing NO; otherwise NO uptake by healthy tissue would reduce the overall NO emission from the plant. In our case, we have always used heavily inoculated leaves (Mur *et al*. 2005*a*, *b*, 2008, 2011). We suggest that wherever possible, experimenters seeking to measure NO from the gas phase should maximize the proportion of plant material producing NO. In passing, these observations have implications regarding NO generation.

## Fermentation products during low oxygen conditions

Under normal aerobic conditions (21 % O_2_), plants produce energy by stepwise oxidation of glucose to the final products CO_2_ and H_2_O (respiration). These steps include conversion of glucose to pyruvate, subsequent oxidation through the tricarboxylic acid cycle to CO_2_, and oxidative phosphorylation, in which atmospheric O_2_ is used to produce H_2_O. Under a lack of O_2_, an alternative process occurs to provide the energy required to sustain the functions of life; pyruvate is converted into acetaldehyde, which is quickly reduced to ethanol, i.e. fermentation (Perata and Alpi 1993). The latter pathway, however, renders far less energy per molecule of glucose than respiration. The ‘lost energy’ is actually trapped in ethanol—the end product of fermentation—and could only be released by further oxidation to water and CO_2_. The intermediate state, low oxygen concentrations, gives a delicate balance between respiration and fermentation. As certain plants slow down their metabolic processes under low-oxygen conditions, knowledge of the parameters determining the respiration to fermentation ratio is important.

The metabolic response and adaptation of plants to anaerobiosis have been reviewed extensively (Perata and Alpi 1993; Vartapetian and Jackson 1997; Pesis 2005). Here, we consider two cases: the influence of low-oxygen conditions on post-harvest conditions in fruit and the response of young plants in submergence conditions experiencing low oxygen concentrations.

Low oxygen concentrations are widely used in controlled-atmosphere storage of harvested fruit, e.g. apples, with the goal of prolonging fruit shelf-life (Toivonen 1997; Watada and Qi 1999) via the reduction of the respiration rate and the ethylene biosynthesis during controlled-atmosphere storage. Fermentation occurs in fruits when oxygen flux to respiring cells is reduced below a critical value. In bulky fruits under natural conditions hypoxia can occur during normal ripening, due to impaired gas exchange with the atmosphere. As certain crops are transported and stored under low-oxygen conditions to slow down metabolic processes such as ripening, knowledge of the parameters determining the respiration to fermentation ratio is of crucial importance for the conservation of the product. High levels of fermentative metabolites may affect the firmness, flavour and colour of the crop. Worldwide post-harvest losses, partly occurring in storage facilities, are estimated to amount to 30 % (Kader 2011).

The effects of ethanol and acetaldehyde on plant tissue under O_2_ deprivation have been widely studied. The high reactivity of acetaldehyde is believed to cause cell death when it is present in relatively high amounts (Monk *et al*. 1987). On the other hand, depending on the concentration, acetaldehyde is believed to inhibit ripening in some crops such as tomato (Beaulieu *et al*. 1997) so that shelf-life can be prolonged by exogenously applying small amounts of this compound (Pesis 2005). In addition, acetaldehyde is known to be one of the (many) components determining the flavour of most fruits.

**Fig. 7:**
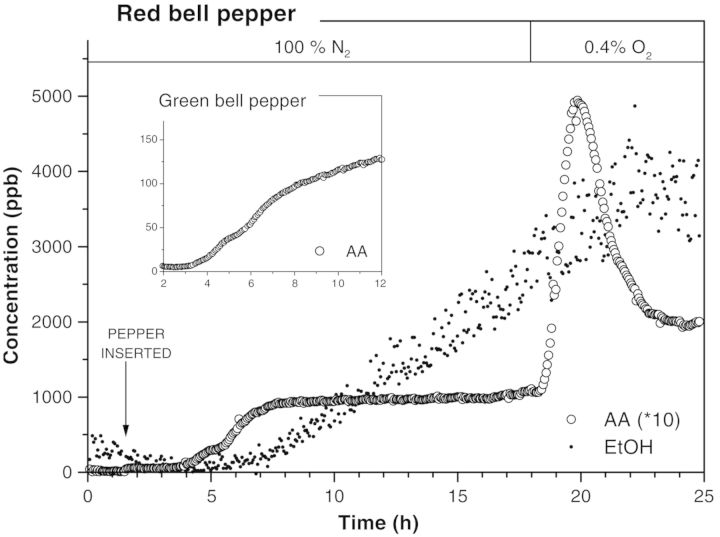
**Acetaldehyde (AA) and ethanol (EtOH) emission of a red bell pepper under zero-oxygen conditions.** The pepper is inserted into the anoxic environment at *t* = 1.5 h. The plateau in the acetaldehyde emission indicates that there is a continuous ongoing fermentation process in which acetaldehyde is the intermediate step and ethanol the final product. Post-anoxic addition of as low as 0.4 % O_2_ leads to a dramatic increase in acetaldehyde emission. In the presence of oxygen, ethanol is enzymatically converted back into ethanol. For clarity, acetaldehyde concentrations have been multiplied by 10 (Oomens *et al*. 1998).

When fermenting plant tissue is transferred from an anoxic to an aerobic atmosphere, an increase in the acetaldehyde emission occurs as a result of oxidation of ethanol accumulated in the tissue during the exposure to anoxia (Pfister-Sieber and Brandle 1994; Boamfa *et al*. 2004). Such a post-anoxic acetaldehyde upsurge appears within a few minutes after transfer to normoxic conditions (Zuckermann *et al*. 1997) (see Fig. 7). This extremely fast reaction strengthened the belief that oxidation of ethanol by rapidly formed active oxygen species, such as hydrogen peroxide (Blokhina *et al*. 2001), stimulated by the enzyme catalase, is responsible for the acetaldehyde formation (Zuckermann *et al*. 1997).

Fermentation also occurs in plants during submergence. Rice, as a staple food and supporting ∼700 million people, is such an example; 16 % of the total area cultivated for rice is potentially vulnerable to flooding. These so-called rain-fed lowland areas often suffer irregular flooding, and losses are severe if submergence lasts for periods longer than about a week (Setter *et al*. 1997; Ram *et al*. 2002). Plant survival of young seedlings depends not only on the depth and duration of submergence, but also on the quality of floodwater. Oxygen and CO_2_ concentrations, turbulence and turbidity are the most important measures of floodwater quality. Oxygen deprivation is caused by an imbalance between the 10^4^ times slower diffusion of gases in water as compared with air, and the oxygen rate consumption by plants. To reduce the impact of oxygen deprivation stress, plants have evolved a wide range of characteristic responses that confer and extend tolerance to anoxia or allow adaptation and acclimation. Next to slowing down the metabolism of the plant, air is transported from the leaves above the water level to the roots via the aerenchyma, and the plant uses underwater photosynthesis to provide the leaves with oxygen (Jackson and Armstrong 1999). The final adaptation is to switch from respiration to the fermentation pathway.

**Fig. 8:**
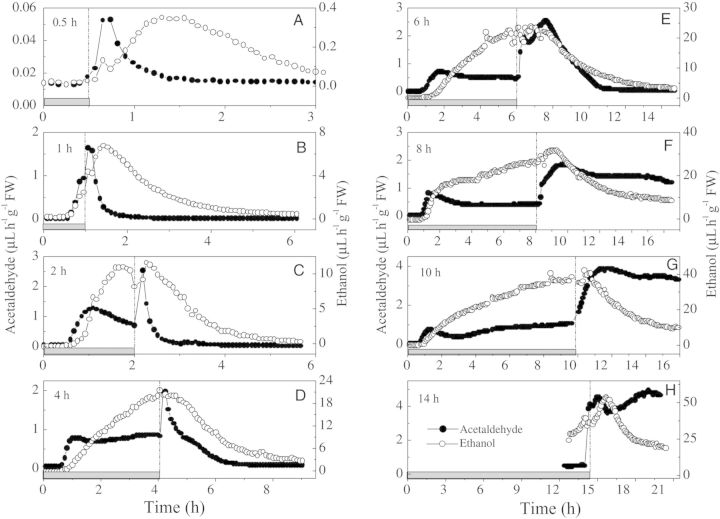
**Effect of anaerobic treatment and subsequent relief from anaerobiosis.** The patterns of ethanol (open circles) and acetaldehyde (filled circles) emissions from single batches of three, 14-day-old, FR13A rice seedlings were measured by online laser photoacoustics. The plants were placed in the dark and given an anaerobic treatment for 0.5 h (A), 1 h (B), 2 h (C), 4 h (D), 6 h (E), 8 h (F), 10 h (G) or 14 h (H) by enclosing them in a cuvette supplied with nitrogen gas flowing at 2 L h^−1^ (grey horizontal bars). Afterwards, the plants were returned to a flow of air (2 L h^−1^) while ethanol and acetaldehyde output continued to be measured (Boamfa *et al*. 2003).

Using laser-based gas detection, the impact of oxygen deprivation on young rice seedling (*Oryza sativa* L.) was investigated and its behaviour on the return to aerated conditions (Boamfa *et al*. 2003, 2005). For this, the dynamics of fermentation/respiration were investigated by monitoring oxygen, ethanol, acetaldehyde and CO_2_ production. Young rice seedlings show clear fermentation under anaerobic conditions and a very specific post-anoxia behaviour, which greatly depends on the duration of anaerobiosis (see Fig. 8). Light can almost completely eliminate fermentation in anaerobic surroundings and also the post-anaerobic or post-submergence peaks in acetaldehyde production (Boamfa *et al*. 2003; Mustroph *et al*. 2006). It minimizes submergence damage by almost completely depressing tissue anoxia by photosynthetically generated O_2_, utilizing respiratory CO_2_.

The kinetics of ethanol and acetaldehyde were also investigated in rice seedlings under micro-aerobic conditions. Various species show different degrees of tolerance to low-oxygen conditions by expressing this in their difference in upsurge of acetaldehyde emission during and after micro-anaerobic conditions (0.05–0.15 %) (Boamfa *et al*. 2005). This release suggests a role for acetaldehyde production in tolerance of rice seedlings under these conditions. The extra acetaldehyde production from ethanol may help to divert the reactive oxygen species away from the damaging lipid peroxidation pathway.

## Ethane as a marker for peroxidative damage

Indirect evidence that reactive oxygen species are present in post-anaerobic tissues of rice has come from results that show release of ethane (C_2_H_6_). Ethane is a marker for lipid peroxidation of polyunsaturated fatty acids from cell membranes by free radicals such as superoxide (O_2_–) (Pfister-Sieber and Brandle 1994; Halliwell and Gutteridge 2007).

Of submerged plants it often said that membrane injury from reactive oxygen species is a post-submergence phenomenon, occurring when oxygen is re-introduced after submergence-induced anoxia.

Using laser-based detection, it was found that ethane emission occurs from rice seedlings which were submerged for several days in the dark, indicating underwater membrane peroxidation and thus severely damaged tissue. In these experiments, submergence-susceptible and -tolerant cultivars were compared, in which higher ethane emissions correlated with observed higher leaf damage and lower survival of the plants (Santosa *et al*. 2007). Seedlings under anaerobic gas-phase conditions produced no ethane until re-aerated: then a small peak was observed followed by a low, steady ethane production. It was concluded that damage during submergence is not associated with extensive anoxia, but that injury is linked to membrane peroxidation in seedlings that are partially oxygen deficient while submerged.

## Biogenic VOCs

Biogenic VOCs (BVOCs) provide important information about various processes in plants, such as the response to stress and signalling to its environment, next to information about the biological pathways of those compounds. On the other hand, BVOCs are also of interest to atmospheric scientists, due to the fact that they can be involved in mechanisms of ozone, aerosol and particle formation, affecting the local chemistry of the atmosphere (Guenther *et al*. 1995; de Gouw and Warneke 2007). Biogenic VOCs, other than CO and CO_2_, consist (primarily) of isoprene and monoterpenes, as well as alkanes, alkenes, carbonyls, alcohols, esters, ethers and acids. Emission inventories show that isoprene and monoterpenes, which belong to the biochemical class of isoprenoids (or terpenoids), are the most prominent compounds (Lichtenthaler *et al*. 1997; Karl *et al*. 2002).

**Fig. 9:**
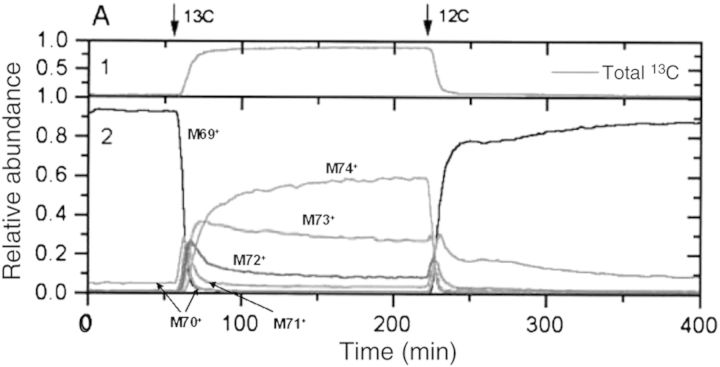
**Kinetics of labelling of oak leaf (*Quercus agrifolia*) isoprene following transfer to a ^13^CO_2_ environment and then back to ^12^CO_2_.** The figure shows PTR-MS data for masses from 69 to 74, which represent isoprene with zero to five ^13^C carbons, respectively. As can be seen, ^12^C isoprene (*m*/*z* 69) disappears rapidly after switching to ^13^CO_2_, giving rise to isoprene molecules up to five ^13^C atoms. This result is in agreement with the understanding that isoprene is synthesized in the chloroplasts. The species with one or two ^13^C atoms disappear rapidly, after which in steady state fully labelled isoprene (*m*/*z* 74) summed only up to 60 % of the detected species. The experiment shows that isoprene synthesis is closely tied to the photosynthesis and photorespiration cycle (Karl *et al*. 2002).

Isoprene (C_5_H_8_) is of interest for atmospheric chemists because of its high reactivity with other gases, and also for plant physiologists because of its light-dependent formation in chloroplasts, which indicates the amount of fixed carbon from CO_2_. Despite the fact that the biochemistry of isoprene formation is known (Lichtenthaler *et al*. 1997), the role of isoprene biosynthesis in plants is still not clear. Isoprene emission represents a significant loss of energy and carbon from emitting plants, and it is assumed that plants must gain some benefits from its synthesis. Researchers hypothesize that plants benefit from isoprene emission because it helps photosynthesis to recover from short high-temperature episodes. The capability of PTR-MS to detect stable isotopes is potentially a very useful tool to study the biological pathways in relation to plant volatiles. Proton transfer reaction mass spectrometry was used to study the formation of ^13^C-isoprene during ^13^CO_2_ fumigation, demonstrating the linkage between photosynthesis and isoprene emission in intact leaves from oak and cottonwood (Karl *et al*. 2002). The advantage of using PTR-MS in labelling experiments is its fast response, online capability and the ability to distinguish simultaneously unlabelled, and up to five ^13^C-labelled, isoprene molecules. ^13^C-labelling allows a detailed analysis of the kinetics of isoprene (Fig. 9).

## Volatile organic compound emissions after leaf wounding and drying

**Fig. 10:**
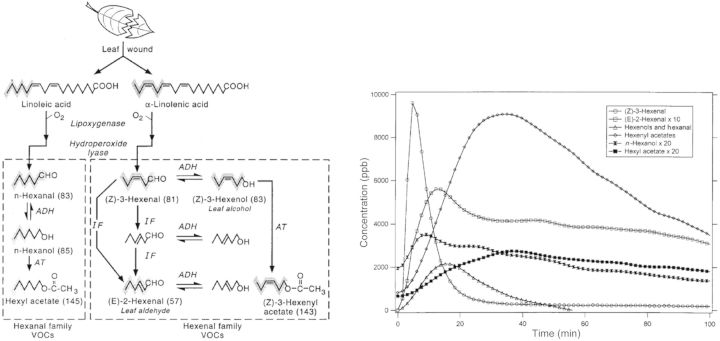
**Emission of green leaf volatiles.** Left panel: scheme for the formation of hexanal and hexenal family VOCs in leaves following wounding. The origins of the six-carbon skeletons of hexanal and hexenal family VOCs from unsaturated fatty acids are indicated with hatched grey. For most of these C6 VOCs, the unique or major positive ions seen in PTR-MS are shown in parentheses. ADH, alcohol dehydrogenase; AT, acetyltransferase; IF, isomerization factor. Right panel: hexyl and hexenyl family VOCs produced from wounded aspen leaves.

Most plants have a similar response to artificial wounding and drying. When considering VOC emission patterns, changes in both the composition and intensities of the emissions can be expected, as compared with non-stressed conditions. These compounds can be newly produced or produced at increased levels. Common compound emission indicates that these compounds occur as a general response to stress in plants and that they have connected metabolic pathways. When a leaf is wounded, the oxidative cleavage of membrane fatty acids (e.g. linoleic and α-linolenic acid) releases a series of aldehydes and alcohols containing C6 compounds (six carbon atoms) and derivates, known as the hexanal and hexenal families (see Fig. 10; Fall *et al*. 1999). The physiological rationale for the rapid formation of these compounds after wounding is that they have antibiotic properties and inhibit the invasion of bacteria and other microorganisms into damaged tissues (Croft *et al*. 1993). In Fig. 10 the emission of VOCs from the hexanal and hexenal families is shown after wounding of aspen leaves (Fall *et al*. 1999). A rapid peak of mass (Z)-3-hexenal emission is observed. This behaviour is consistent with the role of (Z)-3-hexenal as the precursor in the degradation process (see Fig. 10). The rapid decline in (Z)-3-hexenal takes place simultaneously with the rise of other hexenyl derivates. The detection of hexanal is complicated by the lack of unique fragments, but the time evolution of *n*-hexanol and hexyl acetate can be independently observed at a mass to charge ratio *m*/*z* of 83 and 145, respectively. More recently, other studies have also been performed on VOC emission from potted plants and leaves after cutting (Karl *et al*. 2005; Brilli *et al*. 2011).

During drying, similar C6 compounds to those from wounding are emitted by leaves, next to other VOCs. Drying emissions are more intense and longer lasting than the wounding emissions. This could be explained by the decline in water levels that takes place when leaves dry; there will be a point where cellular structures begin to collapse throughout the leaf. This collapse is likely to induce the formation of compounds from the hexenal and hexanal families by the same pathways that occur after leaf wounding, as well as other VOCs. Field drying studies have been performed together with cutting studies above mountain grassland (Bamberger *et al*. 2010), hay field (Karl *et al*. 2001), over grassland (de Gouw *et al*. 1999; Davison *et al*. 2008), alfalfa fields (Warneke *et al*. 2002) and after freeze–thaw wounding (Fall *et al*. 2001). Seco *et al*. (2007) recently reviewed emission and uptake by plants and atmospheric sources, sinks and concentrations.

## Aroma and flavour compounds from fruits

Flavour is one of the most important criteria to define fruit quality. As fruit grows and ripens, a variety of chemical and structural changes take place inside the tissue. Simultaneously, volatile compounds are synthesized, and their composition and concentration will vary during the time course of ripening. Each volatile compound has a huge dynamic range (varying from a few ppbv to several ppmv) during the ripening process; therefore, both concentration level and volatile pattern are good indicators for determining the quality of fruit. The profile of volatile compounds is very complex, including a large diversity of compounds, such as alcohols, aldehydes and esters (Buttery *et al*. 1987; Baldwin *et al*. 1991; Abbott 1999; Goff and Klee 2006).

The ability of PTR-MS to monitor and quantify simultaneously compounds from very diverse chemical groups with high sensitivity and non-invasively makes it an excellent method to monitor aroma-, flavour- and fermentation-related trace gases during growing, ripening or fruit storage. For example, in tomatoes, volatiles associated with flavours described as ‘tomato,’ ‘green’ or ‘grassy’ are derived from linoleic acid (hexanal) and linolenic acid (*cis*-3-hexenal, *cis*-3-hexenol, *trans*-2-hexenal) via lipoxygenase activity (Chen *et al*. 2004). The C6 aldehydes and alcohols derived from free fatty acids are also important constituents of the flavours of a diverse group of plant products including apple, sweet cherry, olive, bay leaf and tea.

Proton transfer reaction mass spectrometry studies provided real-time monitoring of the emission of VOCs during post-harvest aging of berry fruit (Boschetti *et al*. 1999), olives (Vezzaro *et al*. 2011), apples (Boamfa *et al*. 2004; Zini *et al*. 2005), strawberries (Granitto *et al*. 2007), raspberries (Aprea *et al*. 2009) and tomatoes (Farneti *et al*. 2012). Recently, the volatiles emitted by 14 tomato varieties were analysed using multivariate statistics (principal component analysis and cluster analysis) and showed unambiguous separation between varieties. Moreover, a clear fingerprint specific to each tomato type—round truss, cocktail and cherry tomatoes—was identified.

## Plant–herbivore interaction

Ecologists are interested in the role of herbivore-induced volatiles in mediating interactions between plants, herbivores and their natural enemies (De Moraes *et al*. 1998; Kessler and Baldwin 2001). When those interactions occur, changes in both the composition and intensities of the emissions—as with abiotic stresses—can be expected. The defence of plants against feeding herbivores involves direct mechanisms, which include production of or increase in toxic compound levels. Indirect mechanisms can also be involved, such as the release of plant volatile compounds which will attract natural enemies (i.e. parasitoids and predators) of the attacking herbivores. Moreover, those volatiles can also act as ‘intermediates’ in plant-to-plant communication (Baldwin *et al*. 2006).

Using online PTR-MS it was shown that large quantities of VOCs were released when caterpillars were feeding on *Nicotiana attenuata* plants (von Dahl *et al*. 2006) and *Succisa pratensis* leaves (Penuelas *et al*. 2005). The most significant increase was in methanol released by attacked leaves at 24 h after caterpillars started feeding. Methanol production is most likely a result of pectin demethylation in the cell walls and since this process occurs in the apoplast, methanol proves to be a common constituent of the transpiration stream in plants. All other increases in volatile emission rates were much lower than that of methanol, although most of them increased several-fold relative to unattacked plants (including volatile wound compounds and monoterpenes).

## Root emissions

A large variety of VOCs, emitted by the aerial parts of green plants, have been extensively described and characterized (Kesselmeier and Staudt 1999; Dudareva *et al*. 2004, 2006). Induction from root systems received less attention. Although some root exudates have been characterized, especially secondary metabolites and proteins, much less is known about VOCs released by roots. Based on what is known about volatile-induced responses in aboveground plant parts, and the multitude of belowground organisms interacting with the roots in their natural environment, it may be expected that belowground volatile-induced responses are as common as aboveground induced responses.

Proton transfer reaction mass spectrometry was used to study VOC emissions of tree species online (Holzinger *et al*. 2000; Rottenberger *et al*. 2008). As such, emission of methanol, acetaldehyde, ethanol, acetone, acetic acid, isoprene, monoterpenes, toluene and C_10_-benzenes could be identified next to other compounds. Of special interest was a change in the emission behaviour under changing environmental conditions such as flooding or fast light/dark changes. Flooding of the root system caused an up to 20 times increase in several VOCs, dominated by the emission of ethanol and acetaldehyde, which can be explained by the well-described production of ethanol under anoxic conditions of the root system, and subsequent transport and partial oxidation to acetaldehyde within the green leaves (Holzinger *et al*. 2000).

Plant roots create a carbon-rich environment for numerous rhizosphere organisms, including plant pathogens and symbiotic microbes. Proton transfer reaction mass spectrometry was involved in VOC emissions induced specifically as a result of compatible and non-compatible interactions between microbes and insects and *Arabidopsis thaliana* roots (Steeghs *et al*. 2004). Ethanol, acetaldehyde, acetic acid, ethyl acetate, 2-butanone, 2,3-butanedione and other VOCs were found to be produced constitutively regardless of the treatment. Compatible interactions with bacteria (*P. syringae*) and fungus (*Alternaria brassicola*) did lead to a change in the root volatile emissions. Also, when roots were treated with aphids (*Diuraphis noxia*) specific VOCs were released, indicating that this aphid may be a compatible pest for *Arabidopsis* roots.

Roots undergoing herbivore attack were studied using *Brassica nigra* plants infested by cabbage root fly larvae (*Delia radicum*) (Crespo *et al*. 2012). Emitted VOCs were analysed with PTR-MS and gas chromatography–mass spectrometry and showed that several sulfur-containing compounds (methanethiol, dimethyl sulfide, dimethyl disulfide and dimethyl trisulfide) and glucosinolate breakdown products, such as thiocyanates and isothiocyanates, were emitted by the roots in response to infestation. The most typical marker for rapid responses was thiocyanic acid, which is also a prominent fragment of thiocyanates and isothiocyanates. Further investigations showed that the principal compounds emitted after root damage are determined by the plant species, and not by damage type or root glucosinolate composition (van Dam *et al*. 2012). Once they have been determined, the principal compounds may be used as markers for identifying damaged or infested plants. Further analyses of plant enzymes involved in the breakdown of sulfur compounds is needed to reveal the origin of sulfur-containing VOCs from plants.

## Conclusions and forward look

Detection of VOCs released by plants in different conditions can provide valuable information about their physiological processes and the multitude of possible mechanisms of action and signalling, developed by plants in the course of evolution. New methods for online and real-time monitoring of trace gases are available nowadays and have demonstrated their ability in many applications. With the laser-based systems or PTR-MS instrumentation, the dynamics of low concentrations of volatiles are obtained in a time scale of seconds, without an accumulation period that could negatively influence the investigated process. These methods have demonstrated their advantages over the commonly used gas chromatograph in terms of sensitivity and time response. They are indispensable tools for specific applications where their strengths are required and cannot be fulfilled by existing technology, such as gas chromatography.

Significant advances remain to be made in plant research. As an example, hormone signalling and the role of NO are starting to emerge and we believe that online techniques will provide new insights into the understanding of the physiological mechanisms in plants.

## Sources of funding

This work was supported by the GO-EFRO ‘Ultragas—gas analysis systems for quality control of agricultural products and medical diagnostics’ (project no. 2009-010034), the Q-Detect: FP7-KBBE-2009-3 ‘Developing quarantine pest detection methods for use by national plant protection organizations (NPPO) and inspection services’ (project no. 245047) and the EU-FP6-Infrastructures-5 program ‘Life Science Trace Gas Facility’ (project no. FP6-026183). Finally, we thank the Dutch Technology Foundation STW, the Dutch Royal Academy of Science and the Dutch Foundation for Fundamental Research on Matter for their financial support.

## Contributions by the authors

F.J.M.H. and S.M.C. wrote the paper.

## Conflict of interest statement

None declared.
